# Short-Term Efficacy of an Innovative Mobile Phone Technology-Based Intervention for Weight Management for Overweight and Obese Adolescents: Pilot Study

**DOI:** 10.2196/ijmr.7860

**Published:** 2017-08-02

**Authors:** Jyu-Lin Chen, Claudia M Guedes, Bruce A Cooper, Audrey E Lung

**Affiliations:** ^1^ School of Nursing Department of Family Health Care Nursing University of California San Francico San Francisco, CA United States; ^2^ Department of Kinesiology San Francisco State University San Francisco, CA United States; ^3^ School of Nursing University of California San Francisco San Francsico, CA United States; ^4^ Department of Pediatrics North East Medical Services San Francisco, CA United States

**Keywords:** adolescent, obesity, mobile phone technology, website, randomized clinical trial

## Abstract

**Background:**

In the United States, approximately one-third of adolescents are now overweight or obese, and one in six is obese. This financial cost and the larger nonfinancial costs of obesity make obesity prevention and management for adolescents imperative for the health of the nation. However, primary care visits are typically brief, and primary care providers may lack adequate resources to help overweight or obese adolescents to manage weight issues. To augment the efficacy of primary care visits for adolescent weight management, mobile phone technology can be used as an adjunct treatment that provides additional opportunities for encouraging improvement in lifestyle, attainment, and maintenance of healthy weight.

**Objective:**

The purposes of this study were to (1) measure effects of an innovative mobile phone technology-based intervention for overweight and obese adolescents and to (2) examine the intervention’s feasibility for use in primary care clinics.

**Methods:**

The mobile phone-based intervention had three components: use of the Fitbit Flex, participation in an online educational program, and receipt of biweekly text messages during the maintenance phase. A randomized controlled study design was utilized. Data regarding anthropometrics (body mass index [BMI] and waist-to-hip ratio), blood pressure, levels of physical and sedentary activity, diet, and self-efficacy regarding physical activity and diet were collected at baseline and at 3 and 6 months after the baseline assessment.

**Results:**

A total of 40 adolescents participated in the study. At the 6-month follow-up visit, compared to participants in the control group, the mobile phone-based intervention participants had significant improvement in BMI (z=–4.37, *P*=.001), diastolic blood pressure (z=–3.23, *P*=.001), physical activity days per week (z=2.58, *P*=.01), TV and computer time (z=–3.34, *P*=.001), servings of fruits and vegetables per day (z=2.74, *P*=.006), servings of soda and sweetened drinks (z=–3.19, *P*=.001), physical activity self-efficacy (z=2.75, *P*=.006), and dietary self-efficacy (z=5.05, *P*=.001). Medium to large effect sizes were found in these outcome variables.

**Conclusions:**

The use of mobile technologies may offer a practical, reliable adjunct to weight management for overweight and obese adolescents in busy primary care clinics serving adolescents.

**Trial Registration:**

Clinicaltrials.gov NCT 01693250; https://clinicaltrials.gov/ct2/show/NCT01693250? term=Adolescent+ obesity+AND+mhealth&rank=5 (Archived by WebCite at )

## Introduction

During the last three decades, the prevalence of overweight and obesity in adolescents has increased in many parts of the world [1]. Chinese Americans are the largest group of Asian immigrants in the United States and approximately 20% to 25% of Chinese American adolescent are now overweight or obese [2]. At the same body mass index (BMI), Chinese Americans are at higher risk of developing hypertension and cardiovascular disease than are non-Hispanic whites [3,4]. Obesity during adolescent years is associated with many adverse health consequences, including type 2 diabetes mellitus, hypertension, hyperlipidemia, and psychosocial problems [5-8]. Moreover, approximately 70% of overweight adolescents and 80% to 90% of obese adolescents become obese as adults [9]. The direct medical cost of childhood obesity is estimated to be US $14 billion [10]. This financial cost and the larger nonfinancial costs of obesity make obesity prevention and management for adolescents imperative for the health of the nation, especially for high-risk and understudied adolescent populations. Important components in successful weight management for overweight or obese adolescents include increasing physical activity and decreasing sedentary activity and dietary intake [11,12]. Several strategies that promote self-monitoring and setting realistic goals have been found to improve obesity-related health behaviors (eg, physical activity, sedentary activity, and dietary intake) and prevention of obesity [13-15].

Because most adolescents receive their health care in primary care facilities, these settings are an appropriate venue for the development and promulgation of effective, feasible interventions to improve weight status and maintain healthy weight. A recent meta-analysis of 12 intervention studies has reported that a brief office-based primary care intervention to prevent obesity reduced BMI by a small but statistically significant degree (Cohen *d*=–0.04, *P*=.02); in comparison, control groups receiving no treatment, usual care, or active control treatments had no reduction or no substantial reduction of BMI [16]. However, primary care visits are typically brief, and primary care providers may lack adequate resources to help overweight or obese adolescents to manage weight issues. To augment the efficacy of primary care visits for adolescent weight management, mobile phone technology can be used as adjunct treatment that provides additional opportunities for encouraging improvement in lifestyle, attainment, and maintenance of healthy weight.

In the United States, approximately two-thirds of adolescents have a mobile phone, 91% of adolescents use the Internet via a mobile device, and most adolescents have Internet access at home [17]. In an era of advanced, affordable mobile phone technology, mobile phone interventions related to weight management can provide youths with immediate, tailored feedback [18]. The monitor device and apps can also increase adolescents’ ability to understand information, to self-monitor obesity-related behaviors, and to adhere to these clinical recommendations for successful weight management. However, few studies have examined the effects of mobile phone-based interventions used in primary care clinics for weight management in overweight and obese adolescents.

A systematic review of 14 randomized or nonrandomized clinical studies on the effect of technology-based interventions for obesity prevention in adolescents found that only six studies reported decreases in BMI or body fat percentage in the short term (less than 12 months). Results regarding physical activity, dietary behavior, and psychosocial outcomes, found that five of 11 studies did not find improved physical activity, six of 11 studies did not find improved dietary behavior, and two of seven studies did not find improvement in self-efficacy, self-competence, and peer support [19]. However, a second systematic review that examined two randomized clinical trials reported that overweight or obese children and adolescents participated in mobile phone-based interventions of 2 to 3 months’ duration substantially decreased the BMI-z scores compared with children and adolescents in control groups at 12 months after the intervention [20]. These two systematic reviews suggest that in this body of research, efficacy of technology-based interventions on weight management and behavior modification are inconclusive.

In this pilot study, using evidence from research on technology in clinical practice facilitated development of a hybrid intervention that combined lifestyle modification with routine clinical care. Chief among the benefits of this hybrid mobile phone technology-based intervention was the potential to improve health outcomes and reduce obesity in overweight and obese adolescents. This paper describes both phases of the study: phase 1 design and phase 2 pilot study. The aims of the pilot study were to (1) measure effects of an innovative mobile phone technology-based intervention for overweight and obese adolescents and to (2) examine the intervention’s feasibility for use in primary care clinics.

## Methods

### Phase 1: Designing the Intervention

#### Advisory Team and Stakeholders

The first step in the design phase was to establish an advisory team that could engage key stakeholders in this research project. Stakeholder engagement was necessary to ensure that the study would be not only clinically relevant but also feasible for adolescents in primary care settings. An advisory team consisting of two primary care providers and four adolescents was established to provide input on the design of both the study and the mobile phone-based intervention. The research team worked with the advisory team several times to review the study’s purpose and goals and to identify eight topics for an online educational program that we named “iStart Smart for Teens.” Each team meeting lasted approximately 90 minutes. The research team presented evidence-based content that the research team identified and potential ways to present this content to the advisory team. The advisory team worked with the research team to shape this content and also provided ideas on delivery modes that would be attractive to adolescents. The initial mobile phone-based intervention content was developed based on feedback from the advisory team.

#### Adolescent Focus Group Review

The initial version of the mobile phone-based intervention was reviewed by 10 adolescents in a focus group interview. The focus group, which met for 90 to 120 minutes, had two tasks. First, the group identified optimal procedures for using an activity-monitoring device (Fitbit Flex) to track adolescents’ weight-related health behaviors (physical activity, sedentary activity, and food intake). Second, the focus group assessed the appropriateness of the mobile phone-based intervention’s iStart Smart for Teens program. (Both Fitbit Flex and the iStart Smart for Teens program are described subsequently.) In leading the focus group discussions, the moderator used semistructured, open-ended guidelines. Overall, adolescents liked the mobile phone-based intervention. Minor suggestions regarding format of the online module and Fitbit apps were noted in the focus group. Subsequent to the focus group sessions, the research team used the group’s input to refine the online modules. These focus group members did not participate in the intervention pilot study.

#### Structure of the Intervention

**Figure 1:**
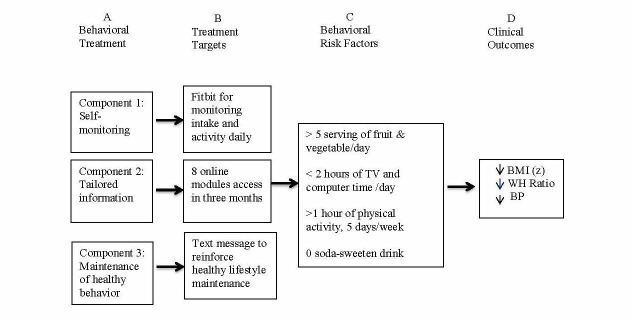
Pathway for Weight Management for Mobile phone-based intervention.

The mobile phone-based intervention had three components: use of the Fitbit Flex (6 months), participation in the iStart Smart for Teens online educational program (3 months), and receipt of biweekly text messages during the maintenance phase (3 months; see Figure 1). Both the Fitbit Flex app and the online program included tracking of physical activity, sedentary activity, and dietary intake progress; setting realistic individualized goals; monitoring progress related to reaching the goals; providing tips for everyday activities; and having interactive apps related to improving weight status and maintaining healthy weight.

#### Fitbit Flex

A commercial monitoring device (Fitbit Flex) and its mobile phone app were selected as the technology for monitoring activity level and dietary intake. The Fitbit Flex is a wristband that tracks steps, distance (of running or walking), calories burned, minutes in activity, and minutes in sleep. Users could also record and track their dietary intake via the Fitbit mobile app. Individuals can check real-time statistics on their cellphone at any time with a quick glance at the Fitbit website or app. Individuals could also use a customized dashboard to analyze data on a daily basis and chart progress over time. Participants were given a study hotline telephone number to call if they encountered problems using the Fitbit Flex and the online program. Participants were asked to wear the Fitbit Flex device and were encouraged to use the app every day for the study’s duration. A weekly message was sent to the adolescents to remind them to use the Fitbit device.

#### The iStart Smart for Teens Program

All information in the program was presented in English. The eight-module iStart Smart for Teens educational program used an online format consisting of short videos and animation narratives; the modules were accessible via both mobile phone and computer. In addition, the mobile phone-based intervention participants received instructions regarding topically relevant activities via mobile phone or computer; supplementary general information and tips were presented via app messages. Each of the program’s modules (ie, classes) could be completed in 10 minutes or less. Participants in the mobile phone-based intervention group were asked to complete one module per week and the entire iStart Smart for Teens program within a 3-month period.

#### Module Structure

In design and style, the iStart Smart for Teens modules were patterned on popular online modules used by the Khan Academy. The Khan Academy online modules use integrated multimedia learning formats and variety of presentation strategies, including animation, short videos, and self-paced learning. The Khan Academy modules’ design and style were suggested for use in the iStart Smart for Teens modules by the pilot study’s advisory board members.

#### Topics

The iStart Smart for Teens program topics pertained to lifestyle modification, weight management, and stress management. The online modules’ titles were (1) Introduction to the Eight Weekly Lessons and the 5-2-1-0 Message (ie, 5 servings of fruits and vegetables, 2 hours of screen time, 1 hour of physical activity, 0 sugary drinks) [21]; (2) Energize Your Health: Energy Balance and Nutrition; (3) Energy Balance: Nutrition; (4) Energy Balance: Physical Fitness; (5) Find Fun in Physical Activity: Energy Out; (6) Less Sit, More Fit: Decrease Screen Time; (7) Smart Problem Solving and Stress Management; and (8) Maintain a Healthy Weight for Life. The program also included activities to enhance adolescents’ self-efficacy and facilitate their understanding and use of problem-solving skills related to physical activity, diet, and coping strategies.

#### Content Sources and References

The content of the iStart Smart for Teens modules was consistent with clinical guidelines for childhood obesity prevention [22]. The modules focused on the “5-2-1-0 message” (to ensure conformity with clinical guidelines, the program adapted information and tips from the We Can! 5-2-1-0 message [21]). The content of the text messages was based on information from several official sources (eg, American Academy of Pediatrics, Centers for Disease Control and Prevention [CDC], and World Health Organization). Text messages and information related to the 5-2-1-0 message included how to be a smart consumer, incorporate colorful and tasty vegetables and fruits in your meals, balanced nutrition for teens, ways to avoid sugary drinks, physical activity for every day, and limited screen time. Tips also included sharing meals with friends so you can enjoy small portions, mixing the colors in your meal by including different vegetables, and going jogging or walking with friends on a beautiful day.

#### Cultural Sensitivity and Appropriateness

Because the majority of patients in the partner primary care clinics were Chinese immigrants, the iStart Smart for Teens modules were tailored to both Chinese and Western cultures. To further ensure cultural concordance, sensitivity, and appropriateness, module materials were modified to reflect common beliefs and practices of Chinese American adolescents living in both Chinese and Western cultures in the United States. Accordingly, the modules discussed common Chinese and Western dietary practices, concepts, and beliefs with regard to promoting balance in health (including the Chinese concepts of *yin, yang,* and *chi*) and presented opportunities for participants to sample healthy Chinese and Western foods.

#### Maintenance Phase

Following completion of the iStart Smart for Teens online program, participants began the mobile phone-based intervention’s 3-month maintenance phase. During this maintenance phase, participants received biweekly text messages that encouraged and stabilized positive behavior changes. The text messages included tips for lifestyle modifications, achievement of healthy weight status, and healthy weight maintenance. Sample messages included “The weather forecast indicates this week is going to be beautiful. Invite friends for a walk or best to catch some balls with friends” and “Got Vegs-add your favorite vegetables to the meal.” Also, the mobile phone-based intervention group participants were encouraged to share their Fitbit data as a basis for weight management discussion and planning with their primary care provider at the clinics. Participants who did not use either Fitbit Flex or the app for more than 1 week received a text message to encourage consistent use. In addition, participants who did not use the Fitbit Flex for more than 2 weeks received a phone call from the research assistant.

### Theoretical Underpinning: Social Cognitive Theory

The design and execution of the mobile phone-based intervention were informed by social cognitive theory (SCT), which holds that several key concepts such as self-efficacy, outcome expectation, skill mastery, and self-regulation capabilities are used to explain and predict behavior [23-25]. Specifically, the intervention’s aim was to increase adolescents’ self-efficacy by setting realistic and achievable goals and outcomes, providing necessary skills, and improving support to promote physical activity and healthy diet [23]. In accordance with SCT, the use of the Fitbit Flex and its apps promoted increased self-monitoring, and the use of the iStart Smart for Teens program provided necessary information and skills for weight-related behavior change (eg, recommendations for fruit and vegetable intake, physical activity, sedentary activity, and sugary drink consumption). The intervention’s text message tips reinforced participants’ adoption and maintenance of healthy lifestyles and weight management practices. Specifically, for overweight and obese adolescents, these tip messages helped to decrease excessive weight gain, waist-to-hip ratio, and blood pressure (see Figure 1).

### Phase 2: Pilot Study of the Intervention

A randomized controlled study design with an active control group was used to estimate the effect size and assess the feasibility of the mobile phone-based intervention. The study was approved by the Committee on Human Research at the University of California, San Francisco (#12-09686).

### Study Procedure

#### Participant Recruitment

To recruit eligible adolescents, the trained research assistant worked with primary care providers at two large community clinics whose patient populations were predominantly Chinese American in northern California. A study invitation letter was posted in the clinics. Pediatricians at the clinics were also given a study invitation letter to give to families of overweight or obese adolescents when they came to the clinics. A response letter and a self-addressed, stamped return envelope was included with the letter. To indicate whether they and their overweight adolescent wished to participate in the study, potentially eligible families were asked to mail the response letter within 2 weeks of receiving it.

#### Eligibility Criteria

To be eligible to participate in the study, an adolescent had to (1) be aged between 13 and 18 years, (2) have a BMI greater than or equal to 85th percentile (as indicated by the CDC growth chart), (3) own a mobile phone, (4) have access to a computer with Internet access, (5) be able to speak and read English, and (6) be a patient at one of the clinics participating in the study. Eligible adolescents also had to be in good health, be free of acute or life-threatening disease, and be able to engage in activities of daily living such as attending school.

#### Induction Into the Study

After receiving signed informed consent forms from both the adolescents and their parents, the adolescents completed online questionnaires regarding dietary intake, physical activity, and self-efficacy related to physical activity at baseline and at 3 months and 6 months after the baseline assessment. The adolescents’ weight, height, waist and hip circumferences, and blood pressure were also measured by a trained research assistant at baseline, 3 months, and 6 months at the study sites. Parents provided demographic data regarding parental age, parental education level, and household income at baseline.

#### Assignment to Group

**Figure 2:**
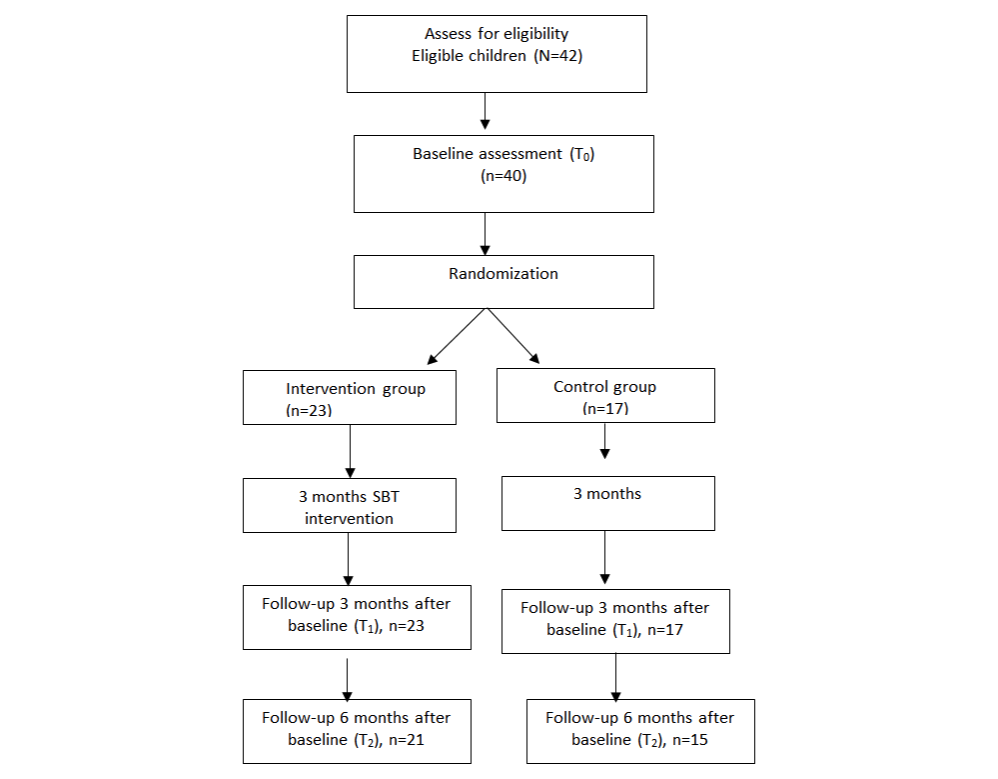
Study CONSORT flow diagram.

After the baseline assessment, the principal investigator randomly assigned eligible participants—40 overweight or obese adolescents—to either the mobile phone-based intervention group (n=23, 58%) or the control group (n=17, 42%) using a randomization table that was stratified by gender; the table was provided by an SPSS program (Figure 2).

#### The Mobile Phone-Based Intervention Group

After completion of the baseline assessments, adolescents in the mobile phone-based intervention group received a Fitbit Flex and downloaded an app and a link to the iStart Smart for Teens program to their mobile phone. Adolescents received in-person demonstrations and written instructions on how to access the Fitbit data and the iStart Smart for Teens program via cellphone and website.

#### The Control Group

After completion of the baseline assessments, control group participants were given an Omron HJ-105 pedometer and a blank food-and-activity diary and were asked to use the pedometer and diary for 3 months. Participants were asked to record and track physical activity, sedentary activity, and food intake in the diary. They also accessed an online program that consisted of eight modules related to general adolescent health issues, such as diet and nutrition, dental care, safety, common dermatology care, and risk-taking behaviors. The online program’s content was based on information from the American Academy of Pediatrics; in the program’s content and presentation, cultural concordance was not a consideration. Completion of each of the online program’s modules required less than 10 minutes. All information was presented in English. All study participants received a US $20 gift card after completion of each data collection.

### Measurement

To determine the effects of the mobile phone-based intervention, participants in both the mobile phone-based intervention group and the control group were measured with regard to anthropometrics (BMI and waist-to-hip ratio), blood pressure (systolic and diastolic), levels of activity (physical and sedentary), diet (fruits/vegetables and sodas/sweetened drinks), and self-efficacy (regarding physical activity and diet).

#### Anthropometry

Anthropometric measurements entailed calculation of BMI and waist-to-hip ratio. Participants’ BMI was determined by dividing body mass by height squared (kg/m^2^). The BMI-z score, which corresponds to the growth chart percentiles, was also calculated. For BMI, adequate sensitivity and specificity has been reported in children and adolescents, with sensitivity ranging from 29% to 88% and specificity ranging from 94% to 100% [26]. *Overweight* is defined as BMI percentile between 85th and 94th percentile; *obese* is defined as BMI percentile greater than the 95th percentile (percentiles are based on data in the CDC growth chart. Waist circumference was measured with a tape measure at the uppermost lateral border of the hip crest (ilium); hip circumference was measured at the maximal protrusion of the buttocks. Measurements were taken twice, and the circumferences were calculated as the mean of the two measurements to the nearest 0.1 cm [27]. The waist-to-hip ratio was derived from the waist and hip circumferences.

#### Blood Pressure

Systolic blood pressure and diastolic blood pressure were measured by using a mercury sphygmomanometer with specific cuff size appropriate for adolescents (Baumanometer, WA Baum Co, Copiague, NY, USA). After participants sat for 10 minutes, blood pressure was measured twice in the adolescent’s right arm; blood pressures were measured to the nearest 2 mm Hg.

#### Physical-Sedentary Activity

To estimate participants’ level of physical activity, they were asked a question from the California Health Interview Survey (CHIS): “Over a typical week, on how many days are you physically active for at least 60 minutes total per day?” [2]. A participant’s stated number of days was used as the estimate for that participant. To estimate participants’ level of sedentary activity, they were first asked to think about their free time during weekdays as well as weekends. They were then asked three CHIS questions: “On a typical day, about how many hours do you usually watch TV or play video games?” “About how many hours per day on Monday through Friday do you use a computer for fun, not schoolwork?” and “On a typical Saturday and Sunday, about how many hours per day do you usually watch TV or play video games?” [2]. The mean of the total number of hours spent daily in these sedentary activities was calculated as sedentary activity time.

#### Fruit/Vegetable and Soda/Sweetened Drinks Consumption

To assess participants’ fruit and vegetable consumption, they were asked two CHIS questions: “Yesterday, how many servings of fruit, such as an apple or banana, did you eat?” Similarly, to assess sugary-sweetened drink consumption, participants were also asked, “Yesterday, how many glasses or cans of sweetened fruit drinks, sports, or energy drinks did you drink?” [2].

#### Physical Activity Self-Efficacy

The five-item subscale of the Health Behavior Questionnaire was used to measure the adolescents’ degree of self-confidence in their ability to successfully participate in various age-appropriate physical activities [28]. Participants were asked if they could perform activities such as “keep[ing] up a steady pace without stopping for 15 to 20 minutes.” Response options were (1) not sure, (2) a little sure, or (3) very sure; higher scores indicated greater self-efficacy.

#### Dietary Self-Efficacy

The self-report dietary self-efficacy questionnaire measured adolescent’s self-confidence in their ability to choose foods low in fat and sugar [29]. The questionnaire contained 15 items, each of which began with the stem “How sure are you...?” Likert scale response options were (1) not sure, (2) a little sure, or (3) very sure; higher scores indicated greater self-efficacy.

### Program Evaluation

After finishing the iStart Smart for Teens program, participants completed a six-item multiple-choice program evaluation questionnaire that asked questions such as “What do you think of the program?” “In what way was this program useful to you?” and “I will recommend this program to other adolescents.”

### Analysis

Demographic characteristics and all major study variables including feasibility data were calculated descriptive statistics. To compare differences between the mobile phone-based intervention group and the control group at baseline, *t* tests were used (when *t* data were normally distributed); nonparametric bootstrapped bias-corrected confidence intervals were used to calculate mean differences between nonnormally distributed variables. Multilevel linear regression models were used to examine between-group differences in outcome variables at baseline and at 3 and 6 months after the baseline assessment. Bootstrap using 5000 draws (to accommodate the small sample size and nonnormality of the outcomes) was used for estimation in the multilevel regression. These models were used to analyze outcomes over time and to ascertain between-group differences in linear change trajectories. Because pilot studies are not designed nor powered to assess the effect of the outcome, conventional *P* values or 95% confidence intervals may not be appropriate because pilot studies are often underpowered to achieve statistical significance at 5%. Alternatively, other *P* values and effect size estimates should be considered [30]. Given that this investigation was a pilot study, a *P* value of less than .10 was set as the statistical significance level.

With 23 participants in the intervention group and 17 participants in the intervention group, we had an 80% chance of detecting a larger effect size (.90) between the two groups as significant at the 5% level (two tailed). Given the purpose of this study was to examine the feasibility and estimate the effect size, analysis focused on the effect size estimate.

To calculate effect sizes, Cohen *d* was used to estimate between-group differences in outcomes at 6 months [31]. Effect sizes were calculated by dividing the difference between the means of the mobile phone-based intervention group and control group by the standard deviation of the baseline scores. Cohen *d* for small effect was 0.2 to 0.49; for medium effect, 0.5 to 0.79; and for large effect, greater than or equal to 0.8 [31]. Data analysis used Stata version 13 (StataCorp LP, College Station, TX, USA).

## Results

### Sample Characteristics

The sample of 40 participants had 23 boys (58%) and 17 girls (42%). Twenty-two adolescents were overweight; 18 adolescents were obese. The adolescents’ mean age was 14.9 (SD 1.7) years. Participants’ mean BMI was 28.3 (SD 4.7) kg/m^2^, and the BMI percentile was 94.0 (SD 3.7). Approximately 90% of the adolescents identified themselves as Chinese American (Table 1). The annual income of approximately 58% of the families was less than US $20,000; the annual income of approximately 95% of the families was less than US $40,000. At baseline, the mobile phone-based intervention group and the control group did not differ in gender, weight status, family annual income, and any other variables (Table 2). The study retention rate at the 6-month follow-up visit was 90% (21/23) for the mobile phone-based intervention group and 87% (15/17) for the control group (Figure 2).

**Table 1:** Demographic characteristics of adolescent participants (N=40).

Variables	Intervention (n=23)	Control (n=17)
Child’s age (years), mean (SD)	15 (1.69)	14.77 (1.60)
Mother’s age (years), mean (SD)	44.32 (4.80)	44.20 (5.45)
Father’s age (years), mean (SD)	46.76 (5.09)	46.43 (5.47)
Mother’s education (years), mean (SD)	10.6 (2.9)	9.3 (3.8)
Father’s education (years), mean (SD)	10.9 (3.3)	9.0 (2.7)
Child’s gender (male), n (%)	14 (58)	9 (53)

**Table 2:** All outcome variables over the three time points (baseline and 3 and 6 months after baseline) by treatment and control groups (N=40).

Variable^a^	Intervention, mean (SD) (n=23)			Control, mean (SD) (n=17)		
	Baseline	3 months	6 months	Baseline	3 months	6 months
BMI	27.37 (3.26)	26.91 (3.25)	26.93 (3.43)	28.35 (4.36)	28.81 (4.43)	29.18 (3.88)
BMI-z	1.60 (0.50)		1.42 (.38)	1.54 (0.42)		1.80 (0.50)
Waist-hip ratio	0.96 (0.05)	0.96 (0.05)	0.96 (0.04)	0.96 (0.05)	0.97 (0.05)	0.97 (0.05)
SBP (mm Hg)	116.70 (9.06)	115.04 (6.96)	114.65 (8.14)	115.65 (18.87)	116.25 (14.88)	115.93 (14.50)
DBP (mm Hg)	72.74 (8.07)	69.39 (7.39)	70.15 (7.53)	69.94 (11.25)	71.63 (9.87)	72.14 (8.71)
Veg/Fruit (serving/day)	3.0 (0.95)	3.91 (1.04)	3.76 (.83)	3.17 (1.24)	3.25 (0.93)	3.08 (0.79)
Soda drink (cup/day)	1.43 (0.90)	0.48 (0.51)	0.35 (0.49)	1.24 (0.97)	1.03 (0.69)	1.07 (0.76)
TV/Computer (hr/day)	3.22 (0.74)	2.25 (0.69)	2.43 (0.60)	3.51 (1.39)	3.59 (1.46)	3.42 (1.57)
PA (day/week)	2.36 (0.99)	3.28 (1.01)	3.09 (1.26)	2.29 (1.57)	1.94 (1.06)	2.25 (1.71)
Nutrition SE	2.39 (0.39)	2.70 (0.40)	2.76 (0.34)	2.58 (0.33)	2.44 (0.30)	2.41 (0.25)
PA SE	2.14 (0.55)	2.75 (0.47)	2.62 (0.51)	2.27 (0.48)	2.28 (0.42)	2.23 (0.43)

^a^ BMI: body mass index; DBP: diastolic blood pressure; PA: physical activity; SBP: systolic blood pressure; SE: self-efficacy.

**Table 3:** Multilevel regression: bootstrap.

Variable		Observation coefficient	SE	z	*P*	90% CI	Effect size *d*^a^
**BMI**							0.62
	Time	0.28	0.10	2.78	.01	0.12, 0.45	
	Group	–1.05	1.22	–0.86	.39	–3.09, 0.95	
	Time×group	–0.58	0.13	–4.37	.001	–0.84, –0.40	
**BMI-z**							0.34
	Time	0.06	0.02	2.4	.02	0.02, 0.09	
	Group	–0.15	0.15	–1.02	.03	–0.40, 0.09	
	Time×group	–0.12	0.03	–4.36	.001	–0.16, 0.07	
**Waist-to-hip ratio**							0.22
	Time	0.01	0.01	0.15	.88	–0.01, 0.01	
	Group	0.01	0.01	0.75	.46	–0.03, 0.01	
	Time×group	0.01	0.01	0.72	.47	–0.01, 0.02	
**SBP**							0.06
	Time	–0.98	0.84	–1.16	.24	–2.36, 0.42	
	Group	0.82	4.71	0.17	.86	–6.93, 8.57	
	Time×group	0.02	0.90	–0.03	.97	–1.45, 1.51	
**DBP**							0.21
	Time	0.93	0.72	1.29	.20	–0.25, 2.12	
	Group	2.21	3.17	0.70	.49	–3.00, 7.42	
	Time×group	–2.66	0.84	–3.23	.001	–4.02, –1.31	
**Fruit/Veg**							0.63
	Time	–0.11	0.17	–0.59	.55	–0.40, 0.19	
	Group	–0.04	0.36	–0.12	.90	–0.63, 0.54	
	Time×group	0.52	0.19	2.74	.006	0.21, 0.83	
**Soda drink**							0.78
	Time	–0.12	0.09	–1.20	.23	–0.27, 0.04	
	Group	0.09	0.27	0.31	.75	–0.37, 0.55	
	Time×group	–0.44	0.13	–3.19	.001	–0.66, –.021	
**TV/Computer time**							0.93
	Time	–0.04	0.09	–0.49	.62	–0.21, 0.11	
	Group	–0.51	0.36	–1.43	.15	–1.10, 0.08	
	Time×group	–0.37	0.11	–3.34	.001	–0.55, –0.19	
**PA**							0.67
	Time	–0.05	0.11	–0.49	.62	–0.24, 0.13	
	Group	0.41	0.38	1.05	.29	–0.23, 1.04	
	Time×group	0.40	0.15	2.58	.01	0.15, 0.66	
**Diet self-efficacy**							0.96
	Time	–0.08	0.04	–1.55	.10	–0.15, 0.00	
	Group	–0.13	0.10	–1.22	.22	–0.32, 0.05	
	Time×group	0.27	0.05	5.05	<.001	0.18, 0.36	
**PA self-efficacy**							0.74
	Time	–0.02	0.08	–0.23	.82	–0.16, 0.12	
	Group	–0.03	0.14	–0.24	.81	–0.27, 0.20	
	Time×group	0.28	0.10	2.75	.01	0.11, 0.45	

^a^ Effect size: small (0.2-0.49), medium (0.5-0.79), and large (>0.8).

### Efficacy of Mobile Phone-Based Intervention

Multilevel linear regression analysis revealed that, at the 6-month follow-up visit, compared to participants in the control group, mobile phone-based intervention participants had substantial improvement of BMI (z=–4.37, *P=*.001), BMI-z (z=–4.36, *P*=.001), diastolic blood pressure (z=–3.23, *P*=.001), physical activity day per week (z=2.58, *P*=.01), TV and computer time (z=–3.34, *P*=.001), servings of fruits and vegetables per day (z=2.74, *P*=.006), servings of sodas and sweetened drinks (z=–3.19, *P*=.001), physical activity self-efficacy (z=2.75, *P*=.006), and dietary self-efficacy (z=5.05, *P*=.001).

Large effect sizes were found in TV and computer time and in dietary self-efficacy; medium effect sizes were found in BMI, physical activity, fruit and vegetable consumption, soda and sweetened drink consumption, and self-efficacy regarding physical activity (see Table 3). A majority of mobile phone-based intervention participants reported using the Fitbit Flex tracking app several times per day and found the app to be helpful.

### Feasibility and Perceived Usefulness of Mobile Phone-Based Program

Seventeen of 23 mobile phone-based intervention participants (75%) reported accessing the Fitbit program via the app or website several times a week, and five adolescents (20%) accessed the program once a week. All the adolescents (100%) who used the Fitbit Flex reported that the device was helpful in tracking physical activity level, and approximately 88% of adolescents found the device helpful in tracking physical activity food intake. All adolescents (100%) in the intervention group would recommend this program to others. The majority of adolescents (91%) shared their Fitbit data with their primary care providers.

## Discussion

This pilot study’s results suggest that the combined use of these tools by overweight and obese adolescents in primary care clinics can potentially improve weight management and the use of a tracking device, its app, and culturally appropriate self-paced online modules is acceptable to adolescents and feasible for use in primary care clinics. Our study’s results substantiate the use of mobile phone technology to prevent excessive weight gain, promote healthy lifestyles, and improve self-efficacy in overweight and obese adolescents. Specifically, we found that overweight and obese adolescents in the mobile phone-based intervention group had (1) increased physical activity and consumption of fruits and vegetables; (2) decreased BMI, diastolic blood pressure, TV and computer viewing time, and soda and sweetened drink consumption; and (3) strengthened self-efficacy regarding both physical activity and diet.

The positive outcomes reported for the mobile phone-based program were due to several factors, three of which were essential. First, the program’s technologies were appropriate for the nature and purposes of the intervention. The use of communication technologies has become an integral part of adolescent life [17], and the utility of these technologies (eg, mobile phones, apps, and monitoring devices) to promote positive health-related behavior changes is well established [20]. For example, the review of mobile phone intervention studies mentioned earlier noted the efficacy of this technology for adolescent weight management—both in increasing adolescents’ engagement in weight management interventions and in decreasing adolescents’ rate of dropout from these interventions [20]. Furthermore, these communication technologies have also proven useful for health information collection and transmission that facilitate patient-provider communication and decision making [32]—another important aspect of the mobile phone-based intervention. Also, with both the apps and the user-based iStart Smart for Teens online modules, simplicity of use contributed to accessibility and user acceptance.

A second major factor in the mobile phone-based program’s success was the early involvement of key stakeholders: adolescents, research team members, and health care providers. The value of these stakeholders’ contributions to our mobile phone-based program design corroborated the finding of a recent systematic review of research on adolescents’ use of mobile phones to support chronic condition management. As with our pilot study, the review reported that the involvement of adolescents and clinical experts in program or prototype design increased adolescents’ use of the interventions [33]. In addition, our involvement of health care providers also proved to be essential in the mobile phone-based program’s implementation [34].

Third, the use of the Khan Academy modules as a model for the iStart Smart for Teens online module format and style resulted in the creation of an education program that was effective in presenting content, engaging participant interest, and promoting learning application and knowledge retention [35]. The education module’s effectiveness was also augmented by the use of a variety of presentation strategies (eg, animation and short videos) that had been recommended by adolescents in our research team as being online learning methods preferred by most adolescents.

Traditional individual or group consultations for behavioral change and weight management are typically costly and time consuming. This mobile phone-based intervention can potentially support a large number of adolescents who, for logistical or other reasons, might not be able to participate in a traditional face-to-face weight management program. Results of our study suggest that implementing mobile technologies for weight management and maintenance of healthy weight may have great potential for decreasing the obesity epidemic while simultaneously improving the health behavior of adolescents who are overweight or obese. Moreover, because primary care clinics typically have limited time for consultation with adolescents regarding active lifestyle promotion and obesity prevention, mobile technologies can be used as valuable resources to assist both patients and providers.

Because this pilot study is one of the first investigations to examine the efficacy and feasibility of a mobile phone-based intervention for overweight and obese adolescents in a primary care setting, the study’s results must be interpreted with caution. The results can only be generalized to similar populations (Chinese Americans). The pilot study’s weaknesses included our use of convenience sampling and self-report measures and the brevity of the follow-up period. Because of the small sample size, we were not able to separately analyze data based on adolescent’s weight status (overweight vs obese). Future studies may need to investigate whether specific subgroup of adolescents can benefit more on this type of program. Use of the Fitbit depended on self-report; therefore, bias and underestimation for the use of the Fitbit device may exist. Also, although we did not collect data on the program’s impact on primary care providers’ practice, brief discussions with adolescents in the mobile phone-based intervention group revealed that most adolescents shared their tracking data with their health care providers as a basis for discussion. We are currently conducting interviews with health care providers to explore the experiences of clinical weight management changes following the providers’ use of mobile technology.

The importance of childhood obesity as an imperative among national health care issues is compelling for several avenues of future research in mobile phone-based interventions. Additional research should assess providers’ perceptions of the utility of mobile phone-based interventions in facilitating weight management and should collect data on the effects of these interventions on primary care providers’ practice. Researchers should also conduct comparative investigations to evaluate the relative efficacies of various technologies in application to behavior and weight change.

## Appendix

Multimedia Appendix 1 Website image.

## Abbreviations

BMI: body mass index
CHIS: California Health Interview Survey
SCT: social cognitive theory
