# Supplementary material for: Short-Term Efficacy of an Innovative Mobile Phone Technology-Based Intervention for Weight Management for Overweight and Obese Adolescents: Pilot Study
Source: Interact J Med Res. 2017 Aug 2;6(2):e12. doi: 10.2196/ijmr.7860 (PMC5559653; doi:10.2196/ijmr.7860)
Supplement: Multimedia Appendix 1 [file ijmr_v6i2e12_app1.pdf]

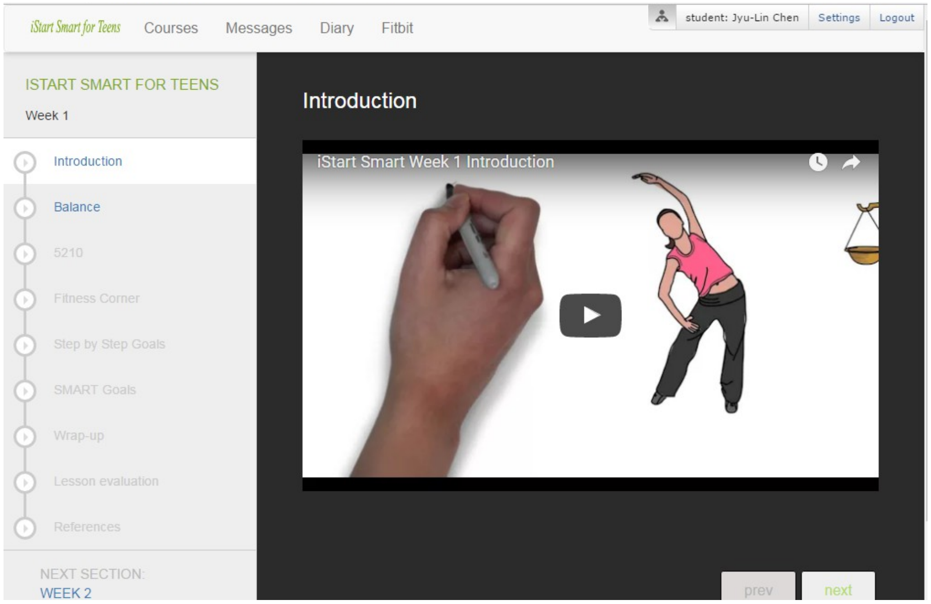
iStart Smart for Teens
Courses
Messages
Diary
Fitbit
student: Jyu-Lin Chen
Settings
Logout
ISTART SMART FOR TEENS
Week 1
Introduction
Balance
5210
Fitness Corner
Step by Step Goals
SMART Goals
Wrap-up
Lesson evaluation
References
NEXT SECTION:
WEEK 2
Introduction
iStart Smart Week 1 Introduction
prev
next

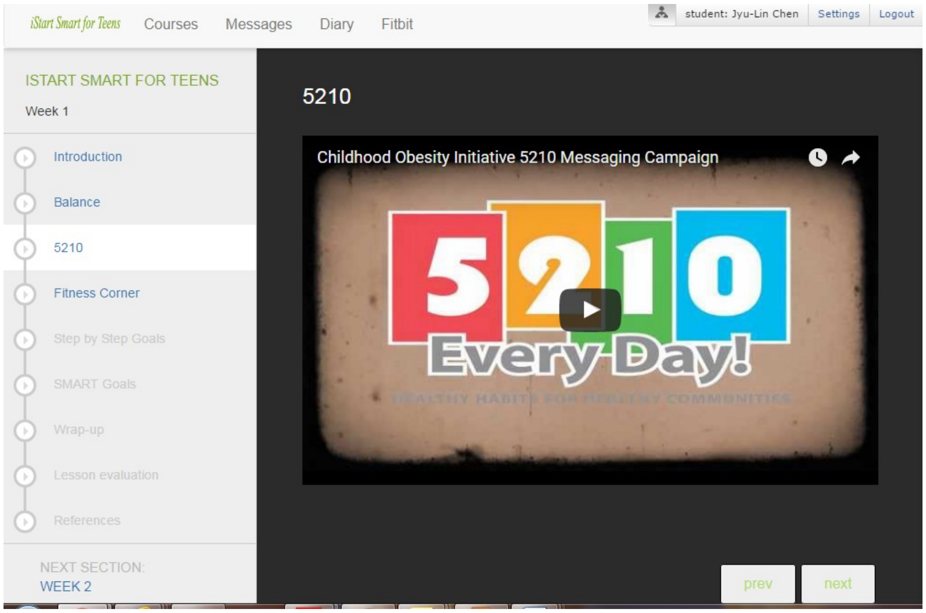
iStart Smart for Teens
Courses
Messages
Diary
Fitbit
student: Jyu-Lin Chen
Settings
Logout
ISTART SMART FOR TEENS
Week 1
Introduction
Balance
5210
Fitness Corner
Step by Step Goals
SMART Goals
Wrap-up
Lesson evaluation
References
NEXT SECTION:
WEEK 2
5210
Childhood Obesity Initiative 5210 Messaging Campaign
5210
Every Day!
prev
next
